# Inheritance and inequality among nomads of South Siberia

**DOI:** 10.1098/rstb.2022.0297

**Published:** 2023-08-14

**Authors:** Paul L. Hooper, Adam Z. Reynolds, Bayarsaikhan Jamsranjav, Julia K. Clark, John P. Ziker, Stefani A. Crabtree

**Affiliations:** ^1^ Department of Anthropology, University of New Mexico, Albuquerque, NM 87131, USA; ^2^ National Museum of Mongolia, Ulaanbaatar, 14201, Mongolia; ^3^ Department of Archaeology, Max Planck Institute for the Science of Human History, Jena 07745, Germany; ^4^ Nomad Science, Ulaanbaatar, 14201, Mongolia; ^5^ Department of Anthropology, Boise State University, Boise, ID 83725-1950, USA; ^6^ Environment and Society, Utah State University, Logan, UT 84322, USA

**Keywords:** economic defensibility, seasonality, property rights, intergenerational transfers, kinship systems, pastoralism

## Abstract

At the headwaters of the Yenisei River in Tuva and northern Mongolia, nomadic pastoralists move between camps in a seasonal rotation that facilitates their animals' access to high-quality grasses and shelter. The use and informal ownership of these camps depending on season helps illustrate evolutionary and ecological principles underlying variation in property relations. Given relatively stable patterns of precipitation and returns to capital improvement, families generally benefit from reusing the same camps year after year. We show that locations with higher economic defensibility and capital investment—winter camps and camps located in mountain/river valleys—are claimed and inherited more frequently than summer camps and camps located in open steppe. Camps are inherited patrilineally and matrilineally at a ratio of 2 : 1. Despite its practical importance, camp inheritance is not associated with livestock wealth today, which is better predicted by education and wealth outside the pastoral economy. The relationship between the livestock wealth of parents and their adult children is significantly positive, but relatively low compared to other pastoralists. The degree of inequality in livestock wealth, however, is very close to that of other pastoralists. This is understandable considering the durability and defensibility of animal wealth and economies of scale common across pastoralists.

This article is part of the theme issue ‘Evolutionary ecology of inequality’.

## 1. Introduction

What principles underlie variation in ownership of, inheritance of, and inequality in wealth? Evolutionary theories of property rights and ownership predict that the stability of ownership should depend on the benefits and costs of maintaining and transferring claims to resources over time [1–6]. According to these principles, the economic defensibility, ownership and inheritance of resources are expected to vary according to time, place and resource type. Local definitions of ownership—also termed property relations [7,8]—are culturally encoded and passed on in the form of traditions, norms and institutions. Patterns of ownership and transmission of wealth between generations are, in turn, key determinants of long-run equality or inequality [9–11].

The use of camps among pastoralists provides a unique opportunity for studying the flexible evolution of property rights and inheritance in humans [12–15]. Whether and how camps are claimed, occupied and inherited varies according to season and other aspects of ecology [16,17]. These patterns can affect the intergenerational transmission of wealth and long-run inequalities [18]. Inheritance patterns also vary according to gender [19]. While patrilineal inheritance is stereotypical among most pastoralists, inheritance through the female line also occurs at some frequency [20,21]. This can result from facultative adjustment to demographic constraints (e.g. unavailability of normative heirs), shared patrilineal and matrilineal residence, and/or normatively bilateral systems of inheritance [22,23].

**Figure 1.**
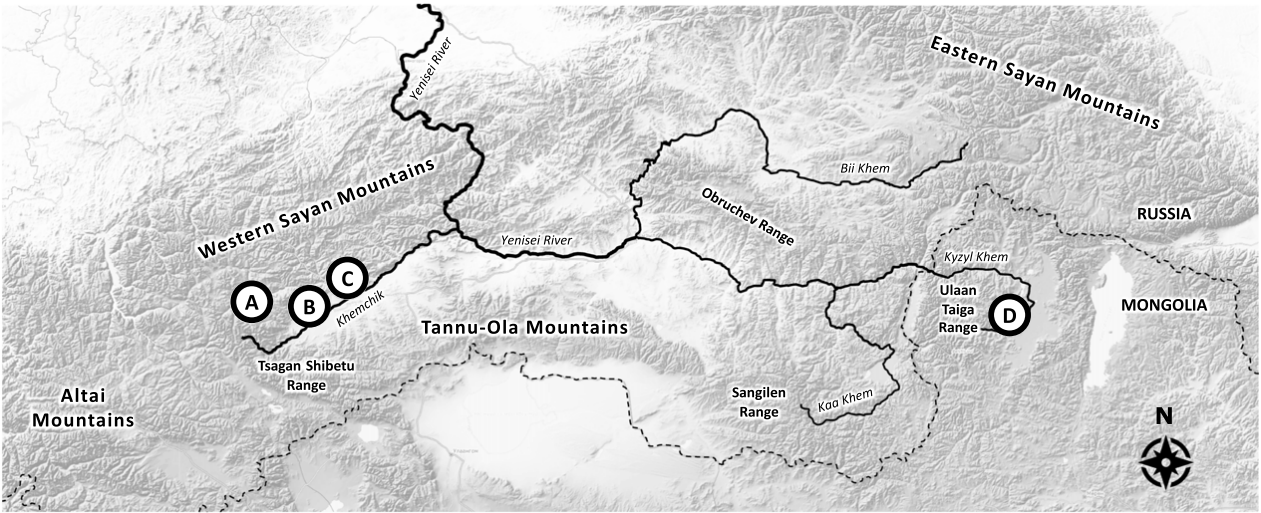
Location of the study sites in the upper Yenisei River basin.

To help shed light on the flexible evolution of property rights, inheritance and inequality, we examine patterns of camp ownership and inheritance among four nomadic communities living within the historical territory of Tannu Uriankhai (figure 1), a region that spans the present-day Republic of Tuva and adjacent areas of the Russian Federation and northern Mongolia [24]. Many studies of Inner Asian pastoralism have highlighted the importance of informal camp ownership within large, commonly or state-owned territories [17,18,25–27]. Here, we focus directly on seasonal and geographical patterns of camp ownership and inheritance in the Khemchik region of western Tuva and the Darkhad region of northern Mongolia. We analyse patterns of camp ownership and inheritance with respect to ecology, season, gender and birth order. We then characterize the intergenerational transmission of wealth, observed degree of inequality and best predictors of pastoral wealth in the sample.

The intergenerational transmission of wealth has been estimated for pastoralists in the Middle East (the Yomut Turkmen of Iran) and in Africa (the Juhaina Arabs of Chad and the Datoga and Sangu of Tanzania) [28]. This study presents comparable estimates for a sample of Inner Asian pastoralists, and provides a window onto the specific mechanics underlying wealth inequality in this unique historical and ecological context.

### (a) Predictions for camp inheritance by season and terrain

Under the Qing, Soviet, and present-day Russian and Mongolian states, Tuvan and Darkhad nomads have negotiated rights to camp sites within a context of dual formal (state-level) and informal (community-level) regulatory institutions [17,24,29]. Within communities, families establish the legitimacy of informal camp ownership based on a history of prior use, inheritance from close kin, or the vouchsafe of other community members [30,31]. During the Soviet period, formal property relations were governed by the collective farm (*kolkhoz*) system [32,33]. During and after the 1990s, the privatization of collective brigades established a new basis for formal, private household property [30,34]. Around the turn of the millennium, familial claims in Tuva were formalized to a greater extent by the registration of camp use-rights with local government offices; in most cases, these registrations documented pre-existing patterns of use, and correspond only approximately with patterns of use in any given year [18,26,30,35].

The effort families put towards maintaining informal ownership and inheritance of camp locations may be expected to vary adaptively depending on season and terrain [12]. Relative to environments with more unpredictable patterns of precipitation (high year-to-year variability), relatively stable patterns of precipitation in the mountainous regions of Tuva and northern Mongolia generally lend themselves to the regular re-use of camps by households over time [36].

The high latitude, high elevation and extreme continental climate of these areas imposes strict constraints on the suitability of camp locations by season. In western Tuva and Darkhad, the predominant pattern of transhumance (seasonal migration) cycles between open steppe or high-altitude pastures in summer, and protected mountainous areas in winter [16,30,37]. During the summer, high-quality pasture tends to be relatively abundant and extensive. Many herders camp in areas of open steppe where the distribution of grass/fodder is relatively homogeneous and geographical circumscription is relatively low [13]. Others occupy high-altitude pastures during the summer that cannot be exploited efficiently during colder months of the year.

Relative to other seasonal camps, access to a suitable winter camp is considered particularly crucial for herd success [18,38]. Because snow builds up on flatter terrain during the winter, the best winter camps tend to be located near south-facing mountain slopes where the sun and wind help to expose underlying pasture [16,39]. In addition to being in relatively short supply, winter camps are also commonly invested with a greater degree of physical capital compared to other seasonal camps [40]. The construction of insulated permanent shelters and cabins at winter sites has been typical of Tannu Uriankhai since at least the nineteenth-century, and intensified further during the Soviet period [16,17]. Hay fields for preparing winter fodder are also commonly located near winter sites, which reinforces their capital value. In the warmer seasons, the nomads occupy mobile yurts and install open pens which are generally less capital intensive than the permanent shelters found at winter camps [16].

The relative scarcity and capital-intensive nature of winter sites together imply greater returns to claiming and bequeathing ownership of winter camps compared to other seasonal camps [18,41]. Mountain pastures are additionally expected to have high economic defensibility as a result of circumscription by steep and forested terrain. This is consistent with the observations of the Russian ethnographer Ermolaev in nineteenth century Tannu Uriankhai:

Open pasture was always regarded as common ground, but the winter pastures, particularly in years when the grass crop was poor, were for individual use: ‘This is my mountain, that is yours', although observance was not necessarily very strict. ([16] p. 83)

Considerations of relative shortage, capital investment and economic defensibility lead to the following set of predictions regarding the inheritance of camps by season and terrain:

P1: winter camps will be more frequently inherited than other seasonal camps;
P2: summer camps will be less frequently inherited than other seasonal camps;
P3: camps in mountainous terrain will be more frequently inherited than camps in other terrains; and
P4: camps on the steppe will be less frequently inherited than camps in other terrains.

### (b) Predictions for camp inheritance by gender and birth order

Data on the inheritance of camps allow analysis of inheritance with respect to kinship. Kinship ties are key resources for establishing community acceptance of claims to camps [30]. Like most other Turkic and Mongolian groups, Tuvan and Darkhad nomads are characterized as patrilineal and patrilocal [36,42]. Despite this patrilineal categorization, several factors lead to expectations of somewhat more balanced, bilateral patterns of inheritance. As Kazato describes in Mongolia, ‘in practice, [winter camps] can be inherited by bilateral and even collateral offspring' ([40], p. 12).

There are a number of reasons to expect mixed patterns of inheritance with respect to gender in this sample. Inheritance through the female line is consistent with an observed tradition of independent and socially prominent women among Turkic and Mongolian pastoralists [43–46]. It may result from matrilocal co-residence that occurs despite normative patrilocality. As Humphrey & Sneath wrote, ‘although in Inner Asia as a whole virilocal residence after marriage is the ideal norm, the Tuva data show that the norm is not always followed' ([36], p. 154). Household co-residence and inheritance are likely to respond flexibly to idiosyncratic demographic realities, such as the unavailability of normative heirs. Matrilocality may occur as a form of bride service performed by less wealthy husbands early in marriage [47]. Because multiple children may inherit and use the same camps, patrilineal and matrilineal inheritance are not always mutually exclusive within a family. In short, the use and inheritance of camps by adult daughters may not be as rare as categorically assumed. This leads to the following prediction:

P5: informal ownership of camps will be regularly passed to adult daughters as well as sons, with greater bias towards sons.

We also evaluate patterns of camp inheritance with respect to birth order. The commonly acknowledged Mongolian norm is that the youngest son co-resides with his parents and inherits their property upon their death (i.e. ultimogeniture) [40,41,47]. Among the Kyrgyz of the Wakhan Corridor in Afghanistan, on the other hand, pastures are normatively passed to oldest sons (i.e. primogeniture) [48]. Other statements about normative camp inheritance in Tuva and Mongolia are not explicit with respect to birth order [16,41,49]. We therefore characterize the frequency of first-born, middle-born and last-born inheritance in the 13 lineages with detailed genealogical data in this sample.

### (c) Predictions for transmission of wealth and inequality

In addition to the inheritance of land rights, we also examine the intergenerational transmission of and inequality in livestock wealth. The extent to which wealth persists within families across generations is quantified by the coefficient of intergenerational transmission, *β* [50]. This coefficient reflects the elasticity of offspring wealth with respect to parental wealth within families. Long-run inequality results from the accumulation of wealth within lineages owing to intergenerational transmission and other factors [9,10,51]. We use the Gini coefficient to quantify observed inequality in livestock wealth across households in this sample. The Gini coefficient—which ranges between 0 and 1 and measures the relative mean difference in wealth between families—is commonly used to compare wealth inequality across past and present societies [52,53].

Several factors make the pastoralist ecological niche prone to high levels of intergenerational transmission and inequality. Livestock is a classically durable, accumulable and transferable form of wealth that stores value over the long term and thus lends itself to intergenerational transmission. The exponential growth capacity of animal reproduction under favourable conditions also creates economies of scale that can allow well-capitalized herders to accumulate wealth faster than those with smaller herds [28].

Intergenerational transmission of herd wealth among pastoralists in the Middle East and Africa is consistently high, in the range of 0.54–0.96, similar to levels observed among intensive agriculturalists [28]. The Yomut Turkmen of northern Iran—who share the present sample's Turkic/Mongolian cultural origins [54]—showed a transmission coefficient of 0.56 (± 0.17 s.e.). High levels of transmission for pastoralists are also associated with high inequality in livestock wealth, with a mean Gini coefficient of 0.51 and a range of 0.35–0.69 [28]. The Yomut Turkmen showed a Gini coefficient of 0.60 (± 0.04 s.e.). The Gini estimated by Murphy [49] in Khentii province in eastern Mongolia is somewhat lower (0.46) but still within the range of pastoralists reported by Borgerhoff Mulder *et al*. [28].

There are reasons to expect that Tuvan and Darkhad herders would show patterns of intergenerational transmission and inequality similar to those observed among other pastoralists. Like in most herding societies, bequests of stock from parents and other close kin in early life provide the foundation for future growth. The herds of parents and children also experience similar conditions owing to co-residence, as well as inheritance of camps and pasture. Reports from Tannu Uriankhai in the nineteenth and early twentieth centuries evidence high levels of inequality, with the wealthiest herders possessing on the order of 4000–6000 head of livestock [16]. Network-dependent access to goods and services meant that inequalities persisted despite the levelling ethos of the Soviet era; inequalities have also grown in many areas of the former Soviet Union following de-collectivization and privatization, particularly in towns and cities [55].

Other factors temper expectations of high intergenerational transmission and inequality in this sample. Responses to insurrection and political purges during the early Soviet period dismantled the wealth and membership of pre-Soviet elites [17,36,56,57]. Collectivization, capital reallocation and mobilization for warfare under the socialist state in the mid-twentieth century further redistributed wealth and access to land [17,36]. There were widespread losses of stock following de-collectivization in the 1990s and generally weak markets for pastoral products in the post-Soviet period [49]. In other parts of Siberia, inequalities fell after widespread job loss and a return to traditional (pre-Soviet) levelling mechanisms [58–60]. In Inner Asia, periodic severe winter weather events (Tuvan: *chut*; Mongolian: *dzud*) can cause catastrophic loss for rich as well as poor herders, potentially upsetting the stability of household wealth over time [61]. On balance, these considerations lead to the following alternative predictions regarding intergenerational transmission:

P6a: the intergenerational transmission of wealth will be similar to that of most other pastoralists (*β* ∼ 0.7); or
P6b: the intergenerational transmission of wealth will be less than that of most other pastoralists (β≪0.7).

They similarly generate the following alternative predictions regarding inequality in livestock wealth:

P7a: inequality in wealth will be similar to that observed for other pastoralists (Gini ∼ 0.5); or
P7b: inequality in wealth will be less than that observed for other pastoralists (Gini ≪ 0.5).

We test each of these predictions below. As a final step, in order to shed light on the basis of the observed inequality, we describe how livestock wealth relates to camp inheritance, non-pastoral wealth, wages and education in this sample.

## 2. Material and methods

Data were collected in collaboration with households in four nomadic communities at the headwaters of the Yenisei River in 2013, 2015 and 2016 (figure 1 and table 1). Interviews were conducted with household heads recording the location of seasonal camps occupied within the last year; whether camps were inherited and from whom; the quality of grasses for animals at each camp; the number of cattle, sheep, goats, horses, yaks, *haynak* (yak-cattle hybrids) and camels owned; an inventory of material wealth (e.g. vehicles, mobile phones etc.); and the age, education and market wages of household members [62]. Interviews were conducted in Tuvan or Mongolian with the help of multilingual research assistants. Research at site D was carried out as part of the Northern Mongolia Adventure and Discovery in Science (NOMAD Science) Project.

**Table 1.** Description of the study sites.

	**site A**	**site B**	**site C**	**site D**
*location*	Tuva	Tuva	Tuva	Mongolia
*province*	Bai-Taiga	Barun-Khemchik	Barun-Khemchik	Khövsgöl
*ethnolinguistic groups*	Tuvan	Tuvan	Tuvan	Darkhad, Tuvan
*mean elevation*	1485 m	919 m	1300 m	1626 m
*year of data collection*	2015	2015	2013	2016
*n households*	13	15	10	57
*n individuals*	62	83	53	240

While reindeer herding is common in some parts of this region [14,63], and some older adults at site D reported familial descent from reindeer herders living in the area in the early twentieth century, there were no reindeer herders living at the sample locations during the time of data collection. Most families maintain a dozen or more sheep and goats; a number of cattle (particularly milk cows for dairy production); and a few horses for transportation. Hooper [62] showed that wealth of better-off herders is reflected predominantly in terms of sheep and goats. Some wealthy herders also specialize in yaks, camels, or horses. The electronic supplementary material, tables S1 and S2 summarize herd composition and other key variables in the total sample and at each site.

For the analysis of camp inheritance, camps were categorized by season of occupation. Camps occupied during the winter with spillover residence during spring or autumn were counted as winter camps, while camps occupied during the summer with spillover residence during spring or autumn were counted as summer camps. Camps only occupied for a single season (autumn, winter, spring, summer) were counted for that season. The terrain characterizing the pasture at each camp was classified as ‘mountain' (mountain slopes, valleys and taiga), ‘river' (in or alongside a riverine floodplain), or ‘steppe' (open, flat grassland). Models predicting the probability that a camp was inherited were estimated using the glm() function in R [64]. The statistical significance of contrasts between seasons and between terrains was bootstrapped using the cluster.bs.glm() function in the clusterSEs package [65]. Because each household contributes multiple camps to the dataset, contrasts included a clustering term for household identity.

For the analysis of livestock wealth transmission and inequality, the total livestock wealth of each household was calculated in units of *bodo*, a traditional measure of livestock accounting employed in Inner Asia [16]. *Bodo* represents a weighted sum of number of adult animals. Pooling sheep and goats together as small stock, 1 *bodo* = 1 cow = 1 horse = 10 small stock = 1 yak = 1 *haynak* = half a camel. The sample included 22 unique parent-offspring pairs (i.e. pairs for which livestock data were available for both the parent's household and the adult offspring's household).

To estimate the coefficient of intergenerational transmission of wealth, *β*—also known as the intergenerational elasticity of wealth—the natural logarithm of parental wealth was regressed on the natural logarithm of offspring wealth using the lmer() function in R [66]. A random effect for the identity of parental household was included to account for the non-independence of multiple offspring from the same parents. Ninety-five per cent confidence intervals were calculated using the confint() function. The Gini coefficient for livestock wealth across households was estimated with bootstrapped 95% confidence intervals using the Gini() function in the DescTools package in R [67]. Owing to the small sample size at each site, this analysis pooled the data from all four sites.

To analyse variation in livestock wealth, education, non-livestock material wealth, wages, age (the mean age of household heads, centred on zero) and camp inheritance (the fraction of camps that were inherited) were regressed on the natural logarithm of livestock wealth using the lm() function. Because of collinearity between these predictor variables, we report the results of bivariate models with each individual predictor as well as a multivariate model that includes all the predictors together. Finally, the relationship between camp inheritance and reported quality of grasses was estimated using the lm() function. The quality of grasses at each camp—reported using a five-level Likert scale that varied from −2 (very bad) to +2 (very good)—was *Z*-scored to have mean of 0 and a standard deviation equal to 1.

## 3. Results

**Figure 2.**
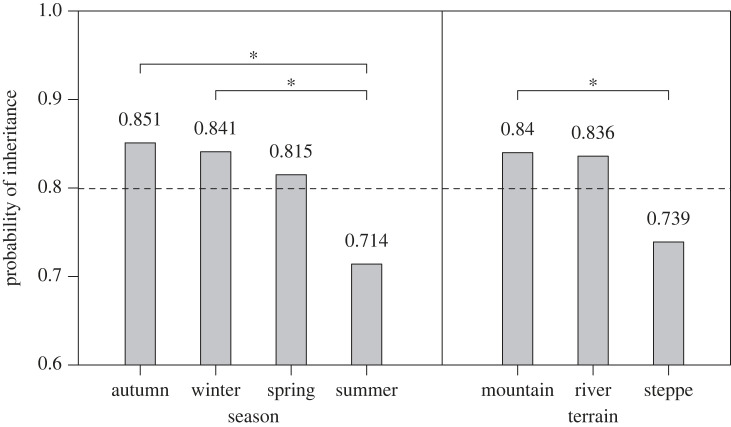
Camp inheritance as a function of season and terrain. Differences marked with an asterisk (*) are significant at the 95% level (*p* < 0.05). The dashed line shows the sample mean of 0.8; *n* = 328 camps.

The mean probability of a camp being inherited in this sample is 80%. The left side of figure 2 shows the probability of inheritance as a function of season. In partial support of P1, winter camps are inherited significantly more frequently (85%) than summer camps (71%). However, autumn camps are inherited just as frequently (85%) as winter camps. In support of P2, summer camps are inherited less frequently than autumn or winter camps. Spring camps are inherited at an intermediate rate (82%) close to the sample average.

The right side of figure 2 shows camp inheritance as a function of terrain. In support of P3 and P4, camps in mountainous terrain are inherited significantly more frequently (84%) than camps on the open steppe (74%). Notably, the inheritance of camps located near rivers are inherited at nearly the same rate (84%) as mountain camps. Unfortunately, co-linearity between season and terrain and small effective sample size makes the independent effects of season versus terrain difficult to identify. The frequency of each terrain type by season of occupation is summarized in the electronic supplementary material, table S3.

**Figure 3.**
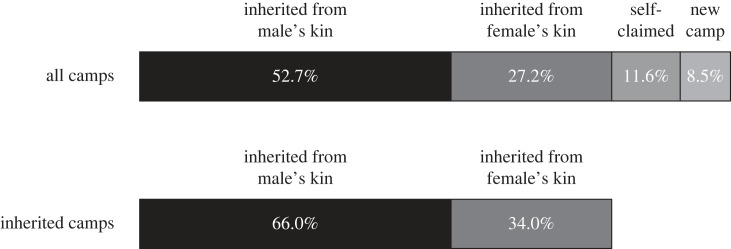
Sources of claims to camps; *n* = 294 camps.

Figure 3 describes the sources of informal ownership of sites. Consistent with P5, the data show a pattern of mixed patrilineal and matrilineal inheritance with a 2 : 1 patrilineal bias. Of camps that were inherited, 66% were inherited from the male head-of-household's kin, while 34% were inherited from the female head-of-household's kin. Of the 20% of camps that were not inherited, 58% were self-claimed and used by the household in prior years, while 42% of camps were newly established in the past year.

There is substantial heterogeneity in the birth order of children that inherit camps. Some of this variation can be understood in terms of life-history stage and the availability/unavailability of inheritors within lineages. Among the 13 families with detailed genealogical data, the four oldest families (those headed by parents over the age of 62; 31% of families) show camp inheritance by youngest sons and out-migration of daughters to their husbands' families or new camps. Three relatively younger families show inheritance to oldest sons (23% of families; in two of these cases, the oldest son was the only potential adult inheritor). Two families showed inheritance to older or middle daughters (15% of families; in one case, daughters were the only available adult inheritors; in the other case, however, the youngest adult son was independent and living with his wife's family). The remaining four families (31% of families) did not yet have inheritors. It can be noted that this subsample is biased towards patriliny in comparison to the larger sample described in figure 3.

Table 2*a* reports the analysis of intergenerational transmission of wealth in livestock. The estimate for the intergenerational transmission coefficient *β* is 0.42. This value is relatively low, about two-thirds of the mean for other pastoralist groups (0.67). Given the small sample size (*n* = 21), however, the confidence intervals for this estimate (0.19–0.64) are wide and overlap substantially with the mean ± 1 s.e. for other pastoralists (0.31–1.38) [28]. This result—a relatively low but imprecise estimate of intergenerational transmission—does not help distinguish between P6a and P6b. In other words, it remains unclear whether transmission is (or is not) exceptionally low here compared with other pastoralists.

**Table 2.** Intergenerational transmission of livestock wealth and inequality.

parameter	estimate	2.5%	97.5%
(*a*) *regression predicting log*(*offspring livestock*)			
intercept	2.381	1.485	3.085
log(parent livestock)	0.415	0.192	0.636
s.d.(parent random effect)	0.581	0.000	1.087
*n* = 21 parent-offspring pairs			
(*b*) *inequality in livestock wealth*			
Gini(livestock)	0.467	0.395	0.556
*n* = 85 households			

The Gini coefficient for inequality in livestock wealth across households in this sample is 0.47 (confidence intervals 0.40–0.56; table 2*b*). This value is very close to the value from Khentii province in Mongolia (0.46; [49]) and the overall mean for other pastoralists (0.51) [28]. Consistent with P7a (and inconsistent with P7b), this suggests that inequality in Tuvan and Darkhad livestock wealth is similar to, not lower than, inequality observed among other pastoralists.

**Figure 4.**
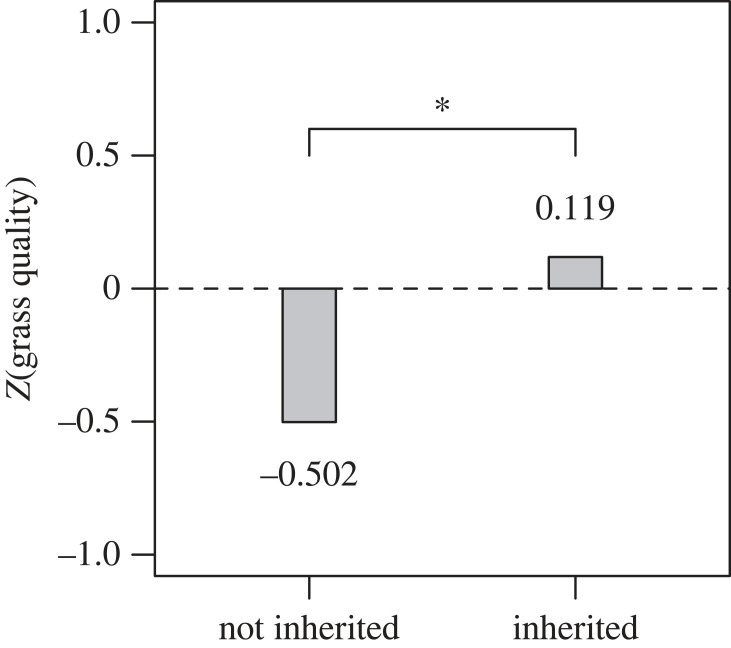
Reported grass quality as a function of whether camps were inherited. The asterisk (*) indicates a significant difference (*p* < 0.001); *n* = 247 camps.

The inheritance of camps is associated with higher reported quality of grasses available for animals (figure 4). It is also associated with reduced expenditures on hay purchased for the winter season (*p* = 0.04). The analysis of predictors of livestock wealth (table 3), however, indicates that inheritance of camps is not a good predictor of present-day livestock wealth. There are also no observable differences in the livestock wealth of families that inherit camps patrilineally versus matrilineally (*p* = 0.40). Livestock wealth instead shows the strongest correlations with education and material wealth outside of livestock (table 3).

**Table 3.** Predictors of livestock wealth.

regressions predicting log(livestock)								
	(*a*) bivariate models				(*b*) multivariate model			
parameter	estimate	2.5%	97.5%	*p*-value	estimate	2.5%	97.5%	*p*-value
intercept	—	—	—	—	0.523	−1.971	3.017	0.676
log(material wealth)	0.281	0.076	0.486	0.008	0.192	−0.047	0.432	0.113
log(education)	0.603	0.111	1.095	0.017	0.369	−0.271	1.010	0.254
log(wages)	0.094	0.011	0.177	0.028	0.041	−0.064	0.146	0.439
age	0.010	−0.005	0.026	0.195	0.005	−0.012	0.023	0.566
camps inherited	−0.056	−0.740	0.627	0.870	0.028	−0.674	0.730	0.936
*n* = 67 households								

## 4. Discussion

This study finds a system of informal camp ownership and inheritance that reflects seasonally and ecologically dependent adaptations. The persistent use of and transmission of rights to camps within Tuvan and Darkhad families makes sense in light of (i) the relatively high economic defensibility of the mountainous and riverine pastures typical of the study areas, and (ii) capital investments made to improve camps, like winter cabins and shelters. Overall, while differences in inheritance are logically patterned according to season and terrain, variance across categories is still quite modest; even the least commonly inherited locations, summer camps, are inherited at a rate of 71%, only somewhat less than the sample mean of 80%.

This case study expands our understanding of the space of possibilities for systems of property rights. It provides another example of a customary system of land-use rights that persists regardless of formal deeds, boundaries or fencing [68,69]. Similar conditions are found among the semi-sedentary hunter–fishers of northern Siberia [58,70,71]. In some ways, the norms governing use rights among Inner Asian nomads are similar to the informal use-based rights (usufruct) systems typical of small-scale horticulturalists. In the horticultural case, however, with the need for frequent and lengthy periods of fallow, claims to the land and its produce tend to be relatively ephemeral [72]. Among Tuvan and Darkhad nomads, by contrast, the benefits of maintaining continuous claims to camps tip the balance towards effectively private, long-term ownership of camps. In this, an emphasis on material capital, and high wealth inequality, these herders have more in common with intensive agriculturalists than horticulturalists [5,73,74].

The substantial frequency of matrilineal inheritance of camps in this sample (34% of inherited camps) is noteworthy. This study contributes to a growing literature reinforcing the obvious importance of female-biased kinship behaviour across human societies [75]. Evolutionary social science will benefit from a more careful examination of the quantitative reality of behaviour directed toward consanguineous and affinal kin, independent of normative classifications [19,76]. More generally, what conditions favour different rates of matrilineal inheritance in the context of normatively patrilineal kinship systems?

Despite a relatively low estimate of intergenerational transmission (*β* = 0.42), the observed extent of inequality in this sample (Gini = 0.47) is very close to that of other pastoralists. This is not surprising: pastoralism has qualities—such as the durability and defensibility of animal wealth, and economies of scale—that promote inequality even in the absence of strong intergenerational correlations [28]. One can note that this estimate, while on par with other pastoralist groups, is still substantially lower than the Gini for household wealth estimated at the national level for the Russian Federation and the USA in 2000 (Gini = 0.7 and 0.8, respectively; [52]).

Ethnographic observation of the households in this sample supports the impression of non-negligible intergenerational correlations in wealth within families. The adult children of the wealthiest herders of the older generation were observably better off in comparison with the adult children of poorer parents. The significantly positive estimate of intergenerational transmission in this sample (with the confidence intervals for *β* ranging between 0.19 and 0.64) validates that impression. What might explain why this estimate is relatively low compared to other pastoralists? The lack of association between camp inheritance and wealth suggests that traditional channels of inheritance may currently be less important determinants of economic success compared to educational attainment and socio-economic resources outside of pastoralism. Weak markets for pastoral goods, limited market access, losses during the Soviet and post-Soviet periods, and opportunities for generating wealth outside the pastoral economy may also have disrupted the signal of transmission between generations.

The economic landscape of rural Inner Asia continues to be dynamic and heterogeneous. While herders depend substantially on economic links to towns and settlements, opportunities for stable employment remain scarce. The adults in this sample have, at different times over the past half-century, been employed as automobile drivers, teachers, lawyers, nurses, mechanics, police, miners and construction workers. Following the breakup of the Soviet Union and the transition from a planned economy, risk-buffering and subsistence needs became predominant in rural Tuva, as in other areas of Siberia [60]. This recent history may be important in interpreting the observed patterns of intergenerational transmission and inequality.

## Data Availability

The data and code associated with this research are available from the GitHub repository: https://github.com/systemsscience/inheritance [77]. The data are provided in the electronic supplementary material [78].
